# Infection-Induced Kidney Diseases

**DOI:** 10.3389/fmed.2018.00327

**Published:** 2018-11-28

**Authors:** Narayan Prasad, Manas Ranjan Patel

**Affiliations:** Department of Nephrology and Renal Transplantation, Sanjay Gandhi Postgraduate Institute of Medical Sciences, Lucknow, India

**Keywords:** infections, nephropathy, glomerular injuries, prevention, glomerulonephritis

## Abstract

Infection induced kidney diseases are of concern for clinicians because timely detection and treatment of infections may cure or limit the extent of injury inflicted by microorganisms causing the infections. Infections can cause kidney injury by either direct invasion, or indirectly by immune mediated mechanisms, which manifest as post-infectious glomerulonephritis, or infection-related glomerulonephritis. Clinical manifestations may be acute or chronic depending on the microorganisms, endemic/epidemic nature and source of infection. All microbials virus, bacteria, mycobacteria, fungus, and protozoa have been implicated in kidney diseases either causing direct kidney injuries or immune-mediated injuries. Infection control practices in large parts of world is limited by poverty, social behavior, high population density, deforestation, inadequate access to safe drinking water, and poor health care facilities. Although, antimicrobials and vaccinations have successfully eradicated and cured many infectious diseases; however injudicious antimicrobial use and emergence of resistant organisms complicated the disease severity like secondary renal amyloidosis with chronic persistent infection. Re-emergence of various infections has been a recent pattern in developed world leading to uncertain diagnostic challenges, and association with kidney diseases.

## Introduction

The spectrum of infection induced kidney diseases is diverse. Infections manifest in form of several renal clinical syndromes: acute kidney injury (AKI), acute and chronic glomerulonephritis syndrome, nephrotic syndrome, acute nephritic-nephrotic syndrome, acute or chronic tubulointerstitial nephritis, and rapidly progressive glomerulonephritis etc. One of the most common presentation is AKI which may occur either *de novo* or on the background of pre-existing chronic kidney disease (CKD). About 40% of patients who recover from AKI have persistent renal dysfunction and many develop CKD (1). In those with pre-existing CKD, infections often accelerate the rate of progression and may lead to end stage renal disease (ESRD).

The mechanisms of kidney injuries with infections are also divergent. Infections cause kidney damage through direct invasion by the offending microorganisms leading to cytopathic injury as observed in pyogenic infections, tuberculosis, leptospirosis, and nematode infestations (2, 3). Infections may also affect the kidneys through immune mechanisms involving microbial antigens that might lead to generation of circulating or *in situ* immune-complexes as in viral glomerulonephritis (GN) or may cause perturbations in innate and cellular immunity as in infection-related glomerulonephritis (IRGN) (1, 3). Kidney injuries may occur as a part of sepsis related multi-organ failure i.e., systemic inflammatory response syndrome (SIRS) with altered cytokine expression, hemodynamic disturbances, hemolysis, rhabdomyolysis, and hepatorenal syndrome (3, 4). Of note, the nephrotoxic effects of antimicrobial agents used for the treatment of infection cannot be undermined which maybe either sole reason or, contributory factor to kidney injury in many conditions (1, 3, 5). We will briefly discuss the viral, bacterial, fungal, protozoan, and parasitic infections induced kidney injuries as well as associated glomerular diseases in the present review. The details of pathogenesis, mechanisms of injury and treatment are beyond the scope of this review.

## Viral infections

A list of viral infections and associated nephropathies is shown in Table 1. Although any acute viral infection can lead to an immune-complex proliferative GN, however the following viral infections are common and induce kidney injuries by various mechanisms including direct cytopathic effects to immune complex mediated GN and vasculitis as well. Hepatitis B virus (HBV), hepatitis C virus (HCV), hepatitis E virus (HEV), human immunodeficiency virus (HIV), dengue virus, and Hantavirus infections can induce glomeruolpathy. Many of the virus infections like parvovirus, Epstein-Barr virus (EBV), and cytomegalovirus (CMV) have been associated with very severe injury in the form of collapsing focal segmental glomerulosclerosis (FSGS).

**Table 1:** Viral infections and associated nephropathies.

**Virus**	**Renal involvement**
**ACUTE**	
Dengue	ATN, ICGN, MesPGN
Hantavirus HFRS	ATN, MesPGN
Varicella-zoster	DPGN
Parvovirus	ICGN, PAN, TMA, HSP
HAV	ICGN, MesPGN, ATN
HBV	ATN, DPGN
CMV	cFSGS, MN, IgA, HSP, ICGN, MPGN, TMA
EBV	ICGN, MN, MsPGN
**SUBACUTE**	
Parvovirus	cFSGS
EBV	cFSGS, MN
HBV	PAN
HCV	PAN
**CHRONIC**	
HBV	MN, Type I MPGN, MPGN1, MC, PAN, IgA, FSGS
HIV	HIVAN, HIVICK, ncFSGS, TMA
HCV	MPGN1, MC,MPGN2, PAN, IgA, MN

*ICGN, immune-complex glomerulonephritis; MesPGN, mesangial proliferative GN; HFRS, hemorrhagic fever and renal syndrome; DPGN, diffuse proliferative GN; PAN, polyarteritis nodosa; PAN, polyarteritis nodosa; TMA, thrombotic microangiopathy; HAV, hepatitis A virus; HBV, hepatitis B virus; CMV, cytomegalovirus; cFSGS, collapsing FSGS; ncFSGS, non-collapsing FSGS; MN, membranous glomerulopathy; HSP, Henoch Shoenlein purpura; MPGN, membranoproliferative GN; EBV, Epstein-Barr virus; MC, mixed cryoglobulinemia; HIVAN, HIV-associated nephropathy; HIVICK, HIV immune complex disease of the kidney*.

### Hepatitis B virus

**Figure 1:**
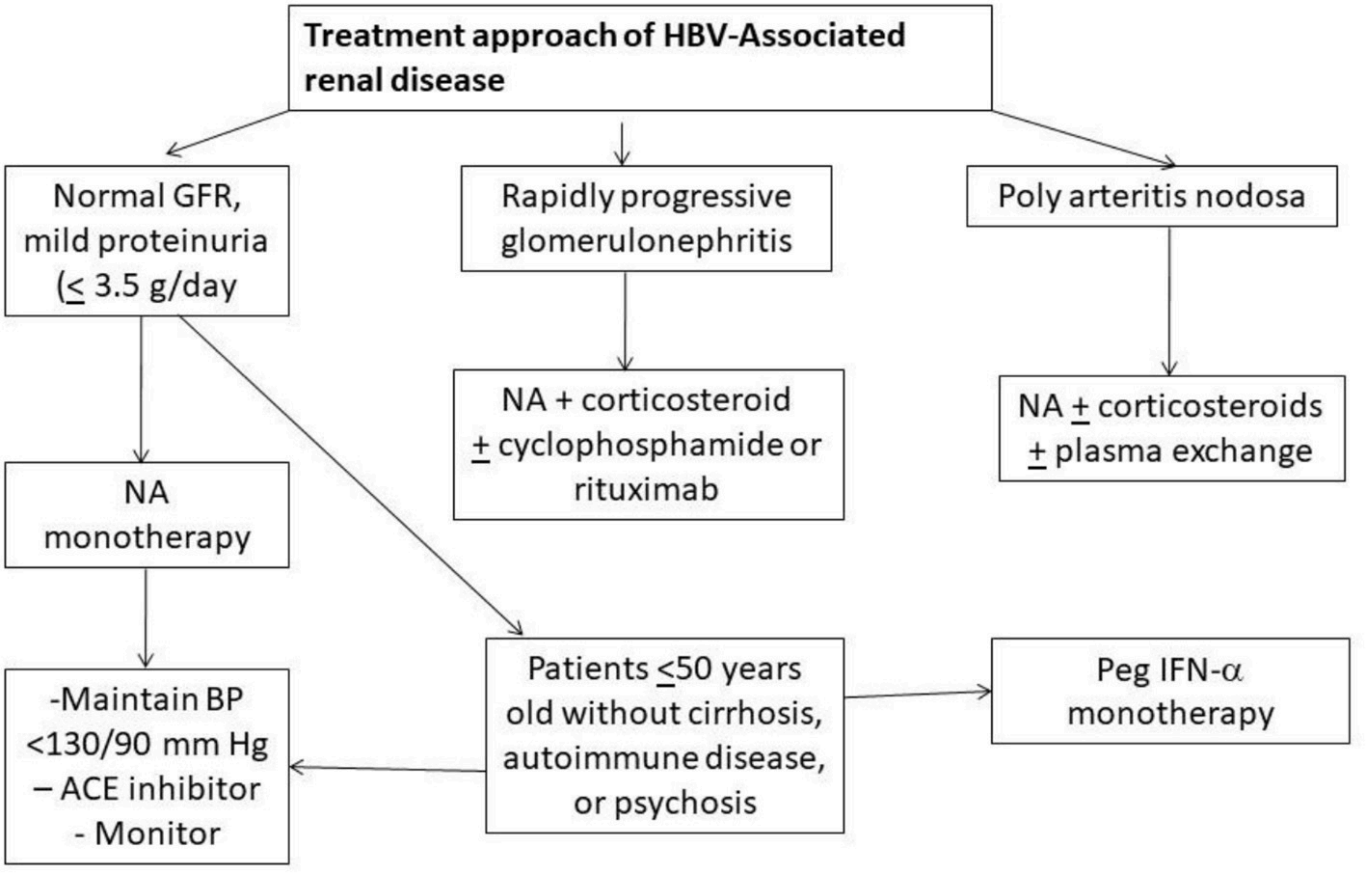
Algorithm showing treatment approach to hepatitis B associated renal diseases (NA, Nucleotide/Nucleoside antagonist); ACE, angiotensin converting enzyme).

Involvement of the kidney is an important extra-hepatic manifestation of HBV infection. With chronic HBV infection, renal disease is observed in up to 3–20% of patients (6). The histologic manifestations of HBV-associated renal disease can be classified as those that occur as a result of either immune-complex glomerulonephritis i.e., membranous glomerulonephritis (MGN), membranoproliferative GN (MPGN), cryoglobulinemic GN and IgA nephropathy (IgAN), or immune complex-related vasculitis i.e., polyarteritis nodosa (PAN) (7). MGN is more common in children whereas mesangioproliferative GN (MesPGN) and IgA deposits are common in adults. Kidney Disease Improving Global Outcomes (KDIGO) recommends the use of interferon or oral antiviral agents that consist of either one of nucleotides (adefovir dipivoxil, tenofovir disoproxil fumarate, tenofovir alafenamide) or nucleoside (lamivudine, entecavir, and telbivudine) reverse transcription inhibitors for treatment of HBV related GN and vasculitis (8). Corticosteroids may be given for a period of < 6 months without a significant effect on liver disease, HBV viremia, or patient morbidity or mortality as long as concomitant antiviral therapy is used (8). An algorithm of suggested approach has been shown in Figure 1 (9). Adefovir and Tenofovir are associated with nephrotoxicity, proximal tubular damage, fanconi syndrome, and osteomalacia. Entecavir is associated with lactic acidosis. Telbivudine is known to cause myopathy, increase creatine kinase, peripheral neuropathy, however may be renoprotective (10). Dose adjustment of drugs according to creatinine clearance should always be kept in mind. Universal vaccination has decreased childhood cases of HBV membranous nephropathy related to horizontal transmission of the virus. HBV infection in renal transplant recipient should be treated with a NA and usually continued for as long as immunosuppressive therapy lasts or at least for 24 months (9, 10).

### Hepatitis C virus

**Figure 2:**
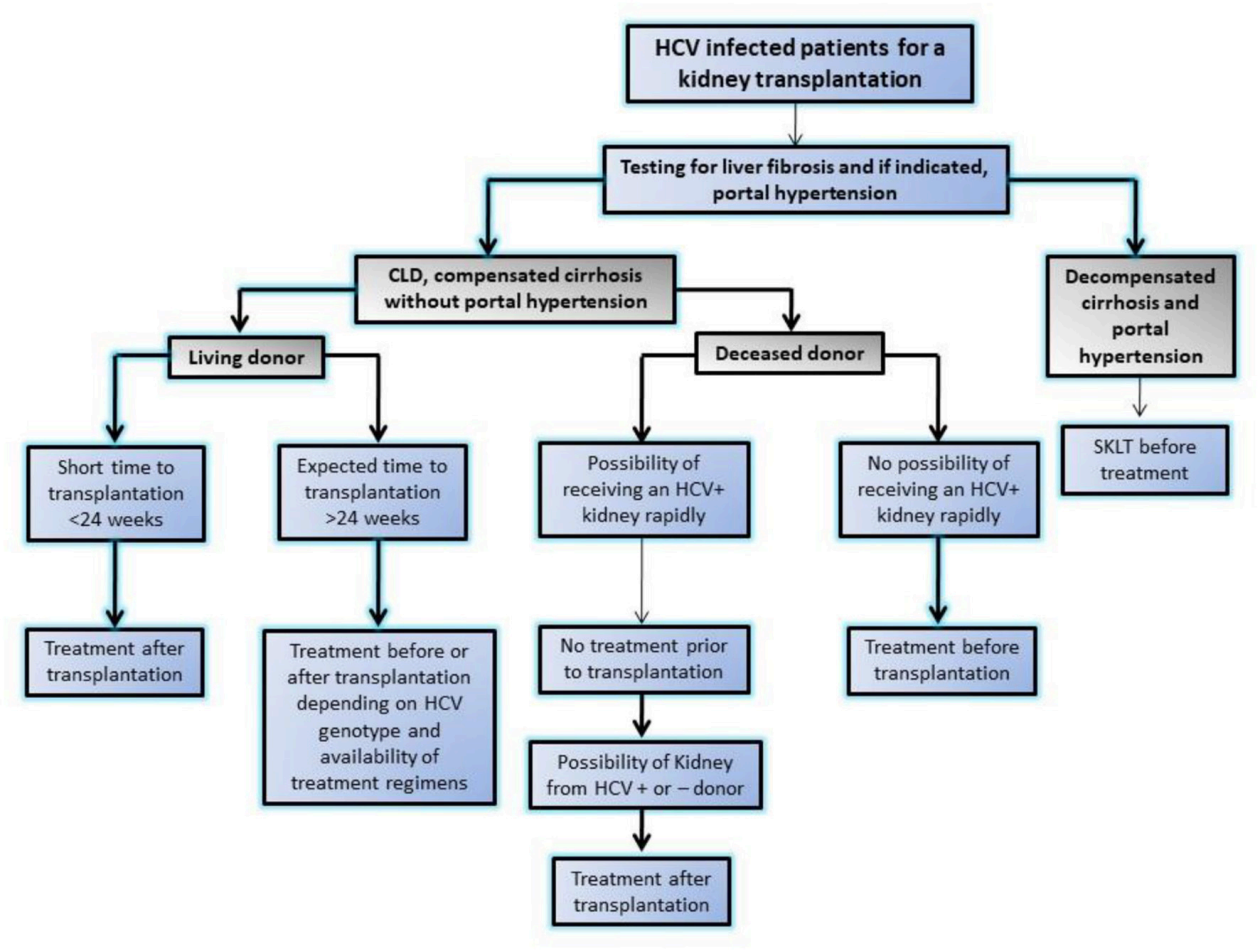
Algorithm showing approach to HCV infected patients for kidney transplantation in modern era of highly effective directly acting anti-viral agents by Kidney disease initiative and global outcome (KDIGO). SKLT, simultaneous liver kidney transplantation.

HCV-associated glomerular disease is primarily due to viral antigen-immune complex formation with their deposition on glomerular basement membrane. The pathologic hallmark is type 1 MPGN with or without type 2 mixed cryoglobulinemia. In addition, immune complex deposition in medium sized blood vessels may lead to PAN similar to HBV (11). Other lesions like MesPGN, focal proliferative GN, and IgAN have also been reported. It is important to note that renal dysfunction due to HCV rarely occurs because of GN (< 10%) and the majority manifest as a consequence of liver disease in form of acute tubular necrosis (ATN), hepatorenal syndrome, and pre-renal azotemia due to the use of diuretics (12). Interferon used to be the mainstream of therapy in the past, but the need for prolonged therapy, poor tolerance, an unsatisfactory sustained viral response and furthermore risk of rejection in renal transplant recipients limited its compliance and use (13). The recent development of potent direct acting anti-viral agents (DAAs) against HCV has enabled successful eradication of HCV with tolerable side effects. The use of DAAs resulted in disappearance of cryoglobulinemia, and resolution of glomerular lesions and are now drugs of first choice in HCV-related glomerular diseases (14). In aggressive disease like cryoglobulinemic vasculitis with impending organ failure, along with control of viremia, immunosuppressive agents (i.e., glucocorticoids, cyclophosphamide, and plasmapheresis) may be warranted to salvage the kidney (15). Wider availability of low cost and generic DDAs in many developing countries is intercepting HCV infections in patients on dialysis and transplantation (16, 17). Recently, KDIGO 2018 guideline (18) emphasized monitoring with nucleic acid amplification test (NAT) in case of HCV infection and also laid down algorithm for the use of DAAs for different specific genotypes and approach for a HCV infected patient for renal transplant with availability of DDAs (Figure 2).

### Hepatitis E virus

Hepatitis E virus infection which was initially thought to be a disease limited to some parts of the developing world only, is now increasingly recognized in many countries of the developed world. HEV infection is reported to be associated with different renal syndromes (19). HEV can cause GN in both immunocompetent and immunosuppressed patients. Many cases of GN which included MPGN with and without mixed cryoglobulinemia, MGN, and IgAN have been described (19, 20). Decline in estimated glomerular filtration rate with HEV infection has been shown in renal allograft recipients. The improvement in renal function and decrease in proteinuria level following HEV clearance, either spontaneously or following therapy suggest the causal relationship between HEV infection and the associated renal injury. European Association for the Study of the Liver has recently formulated a guideline on management of HEV infection in different clinical settings (20).

### Human immunodeficiency virus

Renal diseases in HIV infection manifest in many ways varying from direct invasion by HIV, formation of immune complexes, related to drugs used for treatment, dehydration and various other bacterial, and viral co-infections (21). The demographics of HIV-associated renal disease depend on the population being reported. In the United States and Europe where highly active antiretroviral therapy (HAART) is prevalent, non-collapsing focal segmental glomerulosclerosis (FSGS) is the most common glomerular lesion (19). HIV-associated nephropathy (HIVAN) occurs due to direct viral infection of visceral and parietal epithelial cells characterized by a combination of collapsing FSGS, tubular microcystic dilation, interstitial nephritis, and the presence of intra-cytoplasmic tubuloreticular inclusions without the presence of immune complexes (22). Most patients are HAART naive and of black race with apolipoprotein-1 (APOL1) G1 /G2 variants with various degree of proteinuria and renal dysfunction (23). Median renal survival in patients with zero or one risk allele is lower than two APOL1 risk allele (23). HARRT is effective in reversing renal dysfunction and induce histological regression. Progressive decline in renal function in a patient treated for HIVAN with HAART may be due to various causes i.e., drug toxicity, immune reconstitution syndrome in form of acute interstitial nephritis, immune-complex GN or co-infection with HBV or HCV (24, 25). HIV immune complex disease of the kidney (HIVICK) results from deposition of immune complexes consisting of either HIV antigens or post-infectious immune complexes following other co-infections (23). It represents a pathophysiologic grouping of a wide variety of glomerular diseases i.e., post-infectious GN, membranous GN, IgA nephropathy, MPGN and a “lupus-like” diffuse proliferative GN each with different presentation and prognosis (24). HAART has been effective in some but not all. This may be due to heterogeneous nature of HIVICK and permanent injury to the glomerular basement membrane by immune deposits (26). At present, HAART remains the cornerstone of the therapy in HIV induced kidney diseases. HIV vaccination in future may be step forward in prevention of such diseases.

### Dengue virus

Dengue is a worldwide infection with 40% of the global population living in endemic areas especially Southeast Asia, and Pacific Islands. Infections occur through the bite of the female *Aedes aegyptii*. Dengue is classified into specific syndromes: dengue fever, dengue hemorrhagic fever, and dengue shock syndrome (27). AKI occurs in ~10–33% of patients and is primarily associated with dengue hemorrhagic fever and dengue shock syndrome. AKI results from ATN as a consequence of hypovolemia and capillary leak and/or rhabdomyolysis (28). Glomerulonephritis in dengue is also well-described which may result from immune-complex deposition or, because of direct viral entry into renal tissue (29). Predominant mesangial hypercellularity with immune complexes and IgM deposition or diffuse proliferative pattern is usually the histological picture. The presence of hematuria and proteinuria (both sub-nephrotic and nephrotic) helps distinguish these cases from typical ATN. Treatment strategies remain limited to supportive management in all categories of dengue. Dengue infection in renal transplant recipients may be relatively asymptomatic under effect of immunosuppression; however infection in early period may lead to death (30).

### Hantavirus

Hantaviruses are RNA viruses that belong to the Bunyaviridae family with wild rodent as reservoir. Renal involvement may occur up to 30–40% of cases. Two syndromes can develop: hemorrhagic fever with renal syndrome (HFRS), also called nephropathica epidemica; and Hantavirus pulmonary syndrome (HPS) (31). HFRS manifests clinically with sudden onset of flu like syndrome with fever, myalgia, and headache followed by gastrointestinal symptoms and AKI with oliguria. HFRS leads to renal edema and retroperitoneal leakage of fluid. Acute tubulointerstitial nephritis with mononuclear cells and CD8+cell infiltration is the most prominent finding in the renal histopathology (32). HFRS occurs primarily in Europe and Asia (Old World Hanta) and is caused by the four major Old World Hantavirus serotypes: Hantaan, Dobrava-Belgrade, Puumala, and Seoul viruses. The disease severity and case fatality rate of HFRS varies with the serotype i.e., 0.3% for Puumala infections, 1% for Seoul, 5–10% for Hantaan, and up to 15% for Dobrava. HPS is observed in North-America, Mexico and Panama (New World Hanta) with very high mortality rate up to 30–40% within 24–48 h of admission (32, 33). Hantavirus and other rodent borne disease like leptospirosis have been implicated as one of the potential explanation for Mesoamerican nephropathy (34). No specific therapy is available for Hantavirus infection, management remains conservative and preventive strategies with vaccination are limited as an approved vaccine for Hantavirus infection is still underway.

### Parvovirus

Parvovirus may be associated with three different clinical settings in nephrology practices i.e., glomerulopathy, anemia in ESRD patients and pure red cell aplasia post renal transplantation (35). However, association between parvovirus and glomerular injury is still circumstantial. Viral-induced FSGS has been associated with parvovirus B19. Parvovirus DNA has been identified in renal endothelial and epithelial cells, both visceral and parietal cells, causing collapsing FSGS (90%), idiopathic FSGS (80%), MGN (50%), and minimal change disease (50%) (36). Effective antiviral is lacking, however, Intravenous immunoglobulin may be required in case of red cell aplasia and bone marrow suppression.

### Polyoma virus

Polyoma virus is ubiquitous virus, with seroprevalence of 70–90% in adults, showing reactivation intermittently in both immunocompetent and immunosuppressant individuals (37, 38). Polyoma virus-associated nephropathy (PVAN) is an important infection in patients with renal allograft recipients, affecting 3–10% of patients, causing graft loss in about half of the cases (38). The BK virus exhibits tropism for the renal tubular cells. Immunosupression leads to reactivation of the latent infection and causes graft dysfunction. Viral replication leads characteristic epithelial cell enlargement, karyomegaly, and nuclear inclusion bodies, often associated with an interstitial inflammatory response. Diagnosis is confirmed by immunohistochemistry using an antibody to SV40 large T antigen, and/or electron microscopy showing virions of 40 nm diameter. Monitoring using nucleic acid testing of BKV in blood and urine is recommended for early detection of infection. As there is no specific therapy, mainstay of management of PVAN is reduction of the immunosuppressive therapy and possibly use of various adjuvant medications e.g., cidofovir, leflunomide, and fluoroquinolones (39).

### Other viruses

CMV and EBV can cause acute immune-complex GN and a membranous lesion has been the most common histology reported (34). Respiratory viral infections such as respiratory syncitial virus, influenza, para-influenza, and varicella, have been associated with relapse of MCD (40). Similarly, mumps/measles/rubella vaccines have been linked to a relapse of nephrotic syndrome. In contrast, acute measles, or varicella infection has also led to remission of MCD in many patients (41).

### Bacterial infections

The association of bacterial infection with kidney injuries is diverse. A list of bacterial infections and associated nephropathies is shown in Table 2. Most common ways by which bacterial infections cause renal dysfunction is AKI, which occurs as part of multi-organ dysfunction due to sepsis, SIRS, hypotension, hemolysis, or hepatorenal syndrome (42). Direct invasion of renal tissue by various bacteria either through ascending infection or, through hematogenous spread leads to urinary tract infection, which may lead to renal dysfunction in form of pyelonephritis (2). Occasionally, renal injury may be due to nephrotoxic effects of various antimicrobial agents used as a part of management. Elderly, diabetic, pregnancy, and immunocompromised patients are at increased risk of acute injury and carry high mortality and morbidity (43). As many as 20% of critically ill patient have irreversible renal damage due to acute cortical necrosis and another 40% have incomplete renal recovery, leading to CKD (44). It is not uncommon to observe that episodes of bacterial sepsis accelerate the rate of progression of pre-existing CKD by multiple mechanisms (45).

**Table 2:** Bacterial infections and associated nephropathies.

Bacteria^*^	**Renal involvement**
*Streptococcus pyogenes*	PIGN, IRGN, ATN
*Staphylococcus* (*aureus, epidermidis*)	IRGN, PIGN, DPGN, ATN, AIN, IgA-PIGN, MPGN
*Salmonella (typhi, paratyphi)*	ATN, HUS, AIN
*Escherichia coli*	HUS,
*Leptospira*	ATN, AIN, DPGN, MGN
*Mycobacterium tuberculosis*	CIN, GIN, DPGN, amyloidosis
*Mycobacterium leprae*	MPGN, DPGN, GIN, amyloidosis
*Ligionella* spp.	AIN
*Yersinia enterocolitica*	AIN
*Brucella* species	AIN, ATN, DPGN
*Campylobacter jejuni*	AIN, MesPGN, DPGN
*Corynebacterium diphtheriae*	AIN

* *Any bacteria causing sepsis and multi-organ dysfunction can cause acute kidney injury. Abbreviations: acute tubular necrosis, ATN; PIGN, post-ifectious glomerulonephritis; IRGN, infection-associated glomerulonephritis; DPGN, diffuse proliferative glomerulonephritis; AIN, acute interstitial nephritis; IgA-PIGN, IgA dominant post-infectious glomerulonephritis; MPGN, membranoproliferative GN; HUS, hemolytic uremic syndrome; MGN, membranous glomerulopathy; CIN, chronic interstitial nephritis; GIN, granulomatous interstitial nephritis;MesPGN, mesangial proliferative GN*.

Although, renal involvement in many patients with severe sepsis/SIRS is overt, however infectious agents may induce secondary renal diseases covertly. Many classical renal entities e.g., acute nephritic syndrome, nephritic-nephrotic syndromes, infection induced acute interstitial nephritis and rapidly progressive renal failure in case of IRGN with crescentic transformation etc. may not be a primary glomerular disease, but indirect manifestations of the underlying infection (3, 4). Immune mechanisms induced kidney injury with bacterial infections may lead to acute or chronic interstitial nephritis or glomerulonephritis due to deposition of *in-situ* or circulating immune complex in renal tissue. At one hand the use of immunosuppressive treatment, as frequently used in primary renal disease may threaten the life of the patient by promoting proliferation of the infectious agents while on the other hand, eradication of the infection may lead to healing of the renal disease. Therefore, high level of clinical suspicion, early detection and timely intervention may ameliorate kidney damage in such conditions.

### Post-streptococcal glomerulonephritis (PSGN)

**Figure 3:**
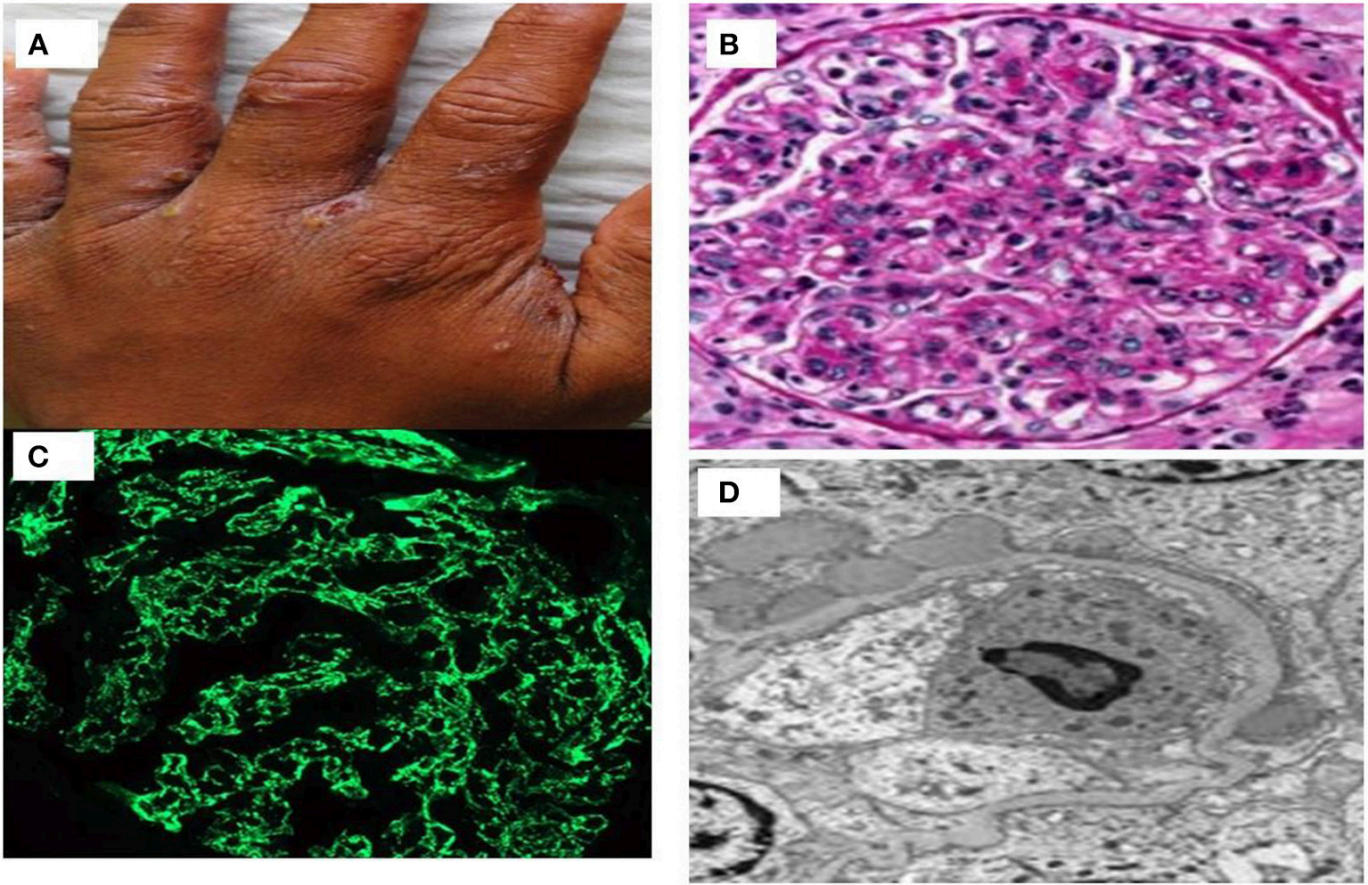
**(A)** A case of streptococcus infected scabies lesion in interdigital web area of hand; **(B)** proliferative glomerulonephritis with neutrophilic exudates; **(C)** immunofluorescence showing starry sky pattern of immunoglobulin deposits; and **(D)** subepithelial humps of immune complex on electron microscopy.

It usually affects children of 2–14 years old. Nephritogenic group *Streptococcus pyogenes* causing either impetigo or upper respiratory infections usually precede nephritis (46). PSGN is rare in industrialized countries and in cadence is also decreasing in developing countries mainly because of improvement in public health, and frequent use of antibiotics. PSGN is an immune mediated glomerulonephritis caused by immune-complex deposits in the subendothelial and mesangial locations consisting of bacterial antigens glyceraldehyde-3-phosphate dehydrogenase (GAPDH) and streptococcal pyrogenic exotoxin B (SPEB) (47). A prototype case of scabies infected with streptococcus who revealed diffuse proliferative GN, starry sky pattern with immunoglobulin deposition on IF study and subepithelial hump on ultrastructural changes on electron microscopy has been shown in Figure 3. Classically, PSGN presents as acute nephritic syndrome with hematuria, hypertension, and edema. Hypocomplementemia may be observed in up to 90% of the patients. Treatment remains supportive as in children and spontaneous recovery is common. Older patients have higher acute complications and have higher prevalence of renal impairment, nephrotic proteinuria, and mortality. Risk factors for the development of CKD are disease onset with nephrotic proteinuria, older age, and diabetic status (48).

### Infection-related glomerulonephritis (IRGN)

It is defined as renal dysfunction due to glomerular inflammation with evidence of ongoing infection at other site. Though incidence has decreased in industrialized countries, it still contributes to about 5% of all glomerular diseases and probably more in underdeveloped areas. Now, it is reported more commonly in adults than in the past; age shift is likely due to improved life expectancy, higher incidence of infections in elderly, and diabetes (49). Other risk factors include alcoholism, malignancy, malnutrition, heart valves, intravenous drug abuse, HIV, and tuberculosis. The sites of infection include skin, upper and lower respiratory tract, heart, oral mucosa, urinary tract, bone and abdominal organs. In adults and elderly, staphylococcal infections are now more common than streptococcal ones. Most common manifestation is acute nephritic syndrome with reduced renal function. Renal histology may reveal MPGN; diffuse proliferative GN, or MesPGN with or without crescents. IRGN has a poorer prognosis as compared to PSGN, more so in adults as compared to children (50). Treatment of IRGN includes identification and eradication of infection and management of complications. Immunosuppressive therapy is not recommended in adults with IRGN. Eradication of infections may be challenging in many situations and a significant proportion of these patients progress to CKD (51). Early detection of infection and its timely treatment may prevent renal involvement.

### Mycobacterial infections

In South-East Asia and Western Pacific Regions, tuberculosis (TB) is still a major public health issue and is expected to pose greater challenges with emergence of multi-drug resistance and co-infection with HIV. Involvement of the genitourinary tract is seen in 6–8% of all cases of extra-pulmonary TB (52). Hematogenous spread of the mycobacterium to the kidney with gradual, asymptomatic progression of the disease leads to delay in diagnosis. Involvement of urinary bladder and ureters leads to obstructive nephropathy. Extensive destructive caseous lesions, ulceration and dystrophic calcification involving renal parenchyma lead to CKD (53). Renal involvement can also present as granulomatous interstitial nephritis that may be difficult to distinguish from sarcoidosis (54). TB is the commonest cause of secondary amyloidosis in the Indian subcontinent (55). Diagnosis of renal TB is usually unsatisfactory due to poor culture techniques, and poor sensitivity of nucleic acid based tests. In late disease, even with effective anti-tubercular drugs renal injury persists and leads to CKD (56).

Leprosy caused by *Mycobacterium leprae* is another important mycobacterial infection of public health problem, which also involves the kidney. Despite reduction in its prevalence, leprosy remains endemic in many parts of the world particularly south Asia (57). In a report of 122 cases from India, reduced creatinine clearance, and proteinuria were common. Autopsy studies revealed a wide spectrum of renal lesions, including renal amyloidosis, glomerulonephritis, tubulointerstitial nephritis and granulomatous disease (58).

## Fungal infections

Fungal infections are not uncommon in hospitalized patients. Major risk factors for fungal infections are older age, female gender, prolonged antibiotic use, indwelling catheter, prior surgical procedures, mechanical ventilation, parenteral nutrition, diabetes mellitusand immunocompromised state including post renal transplantation (59). Most common organisms are *Candida* species albicans as well as non- albicans, and less common fungi are filamentous fungi (*Mucor, Aspergillus, Penicillium*); and rare are endemic fungi (*Blastomycosis, Histoplasmosis, Coccidioidomycosis*). Spread may be ascending (candida) or hematogenous (aspergillus or endemic fungi) (60). Diagnostic tests to differentiate candida colonization from infection have not been standardized. In contrast, presence of filamentous fungi e.g., *Aspergillus* sp., and endemic fungus e.g., *Blastomyces almost* always reflect infection (61). Patients who are symptomatic usually have urinary tract obstruction from masses of fungal elements (fungal balls). Angio-invasion by fungi may lead to numerous renal micro abscesses and extensive renal infarcts leading to renal dysfunction. Treatment with systemic antifungal and surgical removal of the obstructing mass is usually required (62). In extensive diseases despite nephrectomy, mortality remains very high. The prognosis of angio-invasive fungal infection with mucormycosis and aspergillus infections is worse with high mortality (63).

### Protozoal and parasitic infections

There are more than 342 parasites which affects human and at least 20 of them are associated with kidney related ailments. (64) Plasmodia, filariae, leishmania, and Schistosoma are common with diverse epidemiological distribution. The immunogenic and pathogenetic host response to these infections are common in many situations with widely associated spectrum of kidney diseases which extend from AKI to glomerulonephritis, amyloidosis, urologic disorders, and malignancy. We discuss in brief about a few parasitic infections and its association with kidney diseases.

### Malaria

Malaria is one of the most common protozoan infections, which involve kidney. Malaria is endemic in Asia Pacific and Africa, and may be the cause of AKI in 2–39% of such cases in different series (65). AKI is commonly associated with *Plasmodium falciparum infection* and the incidence can be as high as up to 60%; however, kidney involvement may also occur with *vivax, malariae, knowlesi, and ovale infections* (66). Renal injury in malaria infection involves multiple pathophysiological mechanisms. Most common syndrome is AKI, which occurs due to renal ischemia because of hemorheologic changes produced by malaria parasites, volume depletion, intravascular hemolysis, rhabdomyolysis and SIRS produced by these infections (67). Thrombotic microangiopathy associated with *P. vivax* has been reported in different series including ours (68). Malarial AKI is characterized by a rapid increase in uremia. It is often associated with multi-organ dysfunction, severe acidosis, hypoglycemia and hypotension. Artesunate is the antimalarial agent of choice. Aggressive management of complications including renal replacement therapy, hemodialysis, or peritoneal dialysis is essential for successful outcome (69). A small but significant proportion of patients present with glomerular involvement without systemic signs. Acute glomerulonephritis caused by *P. falciparum usually* manifests with mild proteinuria and microhematuria, and uncommonly with nephritic syndrome, however renal dysfunction is rare in this scenario. Renal histology shows mesangial hypercellularity with infiltration of pigment-laden macrophages and parasitized red cells; and endocapillary proliferation as well. A picture similar to HUS with platelet-fibrin thrombi and patchy necrosis may be present in a few biopsies (70). Treatment with anti-malarials has shown to normalize urinary abnormalities. *Plasmodium malariae*, which causes chronic quartan malaria, may be associated with steroid-resistant nephrotic syndrome, known as tropical nephrotic syndrome. Typical presentation is massive proteinuria with normal cholesterol, hypertension, and progressive renal failure. Kidney biopsy shows proliferative pattern with granular deposits of IgG, IgM, and C3, indicating a immune-complex medicated injury (71). Prognosis of the disease is poor as it usually progresses to ESRD even with successful treatment of the malarial infection (72).

### Leptospirosis

Leptospirosis is caused by spirochete *Leptospira* spp., is a zoonosis endemic in tropical climates. Infection is transmitted to humans through animal urine. Due to high sero-prevalance in endemic area, it has been implicated in development of Mesoamerican nephropathy (73). The spectrum of renal injury includes mild proteinuria, urinary sediment abnormalities, tubular dysfunctions, and AKI primarily due to interstitial nephritis (74). Renal involvement is usually non-oliguric AKI as a part of multi-organ involvement, along with pulmonary hemorrhage, and acute respiratory distress syndrome. Antibiotic treatment is mainstay of therapy with requirement of renal replacement therapy for severe renal failure and respiratory support for respiratory distress syndrome. A proportion of survivors show persistent defect in tubular function and reduced renal function (75).

## Leishmaniasis

Leishmaniasis or Kala-azar is caused by *Leishmania* sp. with humans as reservoir and sand fly as vector. Leishmaniasis primarily affects reticuloendothelial system, and renal involvement is associated with visceral Leishmaniasis. It is characterized by fever, malaise, weight loss, hepatosplenomegaly and lymphadenopathy. Renal histology is varied i.e., chronic interstitial nephritis, MPGN, and amyloid deposits. AKI has been reported in children with visceral leishmaniasis treated with systemic antimonial drugs (76).

### Schistosomiasis

Schistosomiasis is endemic in Africa, South America and Far East. Of the infected subjects, 60% are symptomatic and 10% had renal dysfunction (77). Lower urinary tract is the primary site of S*. haematobium* infection. Granulomatous response around its ova produces small “pseudotubercles” in the bladder mucosa which consolidate toform sessile masses and ulcerate. Presentation is usually with microscopic to macroscopic hematuria. Progressive disease leads to fibrosis and bladder calcifications resulting in outflow obstruction or vesicoureteral reflux and finally, chronic pyelonephritis. Moreover, secondary bacterial infection with *Pseudomonas* or *Proteus* is commonly associated with disease (78). Immune complex-mediated renal disease occurs with patients with *S. japonicum* and *S. mansoni* but not with S. *haematobium*. Various glomerular histology i.e., mesangial proliferative, diffuse proliferative, membranoproliferative, focal segmental, and sclerosing lesions result from deposition of immune complexes in the glomerulus. Severe disease is usually symptomatic and progressive even after eradication of infection (79). Immune-mediated tubulointerstitial nephritis with dense interstitial infiltration, fibrosis, and periglomerular has also been described with *S. mansoni* (80). Antiparasitic treatment is very effective in early bladder disease but not in advanced and chronic disease involving kidneys. Urological surgery including urinary stenting may be required for the relief of obstructive nephropathy (81).

Of all the filarial parasites, *Wuchereriabancrofti and Onchocerca volvulus* are associated with renal disease. Transmitted by Culex mosquitoes bite, *W. bancroftiis* is endemic in sub-Saharan Africa and Southeast Asia. Usual clinical syndromes are tropical eosinophilic pneumonia, chyluria with hematuria and elephantiasis (82). Few patients may present with nephritic syndrome and immune complex mediated proliferative GN (83).*O. volvulus* is rarely associated with minimal change disease or chronic sclerosing GN with progressive renal impairment (84). Once established, anti-filarial treatment is ineffective in reversing renal disease.

Scrub typhus is caused by spirochete *Orientia tsutsugamushi*, and transmitted by the bite of trombiculid mite. It is considered as an endemic re-emerging disease in South-East Asia and South-Western Pacific region. It usually presents with acute febrile episodes in endemic regions with multi-organ dysfunction and has high case fatality rate if not diagnosed early (85). Most patients have some subclinical renal involvement and symptomatic AKI has been observed in half of the cases (86). Early detection and treatment with antimicrobials tetracyclines or microlides show rapid response.

## Summary

Evolutions in medicines led to cure of many diseases and infection related diseases are one of them. Anti-microbials and vaccinations prevented human beings from many communicable diseases. Classically, CKD is considered a non-communicable disease (87), and diabetes and hypertension are common etiologies. Chronic glomerular and interstitial diseases are still amongst the leading causes especially in developing and underdeveloped parts of the world (88). Infections by various mechanisms cause spectrum of renal disease ranging from AKI, acute and chronic GN, interstitial nephritis, pyelonephritis and many times obstructive uropathies. Association of various infectious agents has been implicated in many renal syndromes including CKD of unknown etiology (CKDu) reported from various parts of the world (89–91). Many patients with AKI progress to CKD i.e., form asymptomatic urinary abnormalities i.e., proteinuria, hematuria, urinary acidification and concentration defects to ESRD requiring dialysis or renal transplantation (92, 93). In parts of the world where infections remain common in the population, the direct and indirect contribution of infections on development and progression of renal dysfunction cannot be underestimated. Infection control practices in large parts of the world is limited by poverty, social behavior, high population density, deforestation, inadequate access to safe drinking water and poor health care facilities (94). Moreover, injudicious antimicrobial use leading to resistant organisms has implications for disease severity and resultant kidney disease (95). Re-emergence of various infections has been a recent pattern in the developed world leading to uncertain diagnostic challenges and the outcomes (96). Limited data in literature limits an accurate assessment of impact of infections on prevalence of kidney disease. Early detection and aggressive treatment of infections with effective anti-microbial agents may limit the degree of renal injury. Many forms of infectious AKI may be prevented by effective vector control practices. Proper referral and follow-up is imperative to identify those with higher risk of progressive renal disease (94).

## Author contributions

All authors listed have made a substantial, direct and intellectual contribution to the work, and approved it for publication.

### Conflict of interest statement

The authors declare that the research was conducted in the absence of any commercial or financial relationships that could be construed as a potential conflict of interest.
